# One-stage tubeless percutaneous nephrolithotomy for asymptomatic calculous pyonephrosis

**DOI:** 10.1186/s12894-022-00983-z

**Published:** 2022-03-07

**Authors:** Shijie Guo, Xicai Zhang, Fengyue Li, Chunyue Sun, Yonghe Zhang, Xiande Cao

**Affiliations:** 1 Clinical Medical College of Jining Medical University, Jining, 272000 Shandong China; 2 Department of Urology, Affiliated Hospital of Jining Medical University, Jining, 272000 Shandong China

**Keywords:** Calculous pyonephrosis, Kidney stone, Percutaneous nephrolithotomy, Tubeless

## Abstract

**Background:**

In recent years, the safety and effectiveness of one-stage percutaneous nephrolithotomy (PCNL) for the treatment of calculous pyonephrosis have been proven. In order to further reduce postoperative pain and hospital stay, we first proposed and practiced the idea of one-stage tubeless percutaneous nephrolithotomy for calculous pyonephrosis.

**Methods:**

A retrospective analysis was performed of case data of 30 patients with asymptomatic calculous pyonephrosis treated in our center with one-stage PCNL from January 2016 to January 2021. Patients were routinely given 20 mg of furosemide and 10 mg of dexamethasone sodium phosphate injection intravenously at the beginning of anesthesia. Among them, 27 patients successfully underwent one-stage tubeless percutaneous nephrolithotomy, while 3 cases were given indwelling nephrostomy tubes because of proposed second-stage surgery or the number of channels was greater than or equal to 3. All patients were operated on by the same surgeon.

**Results:**

Preoperatively, 11 of 30 patients (8 men and 22 women) had positive urine bacterial cultures, and all were given appropriate antibiotics based on drug sensitivity tests. All patients completed the surgery successfully. The mean operative time was 66.6 ± 34.7 min, the mean estimated blood loss was 16.67 ± 14.34 mL and the mean postoperative hospital stay was 5.0 ± 3.1 days. The mean postoperative hospital stay was 4.6 ± 2.5 days among the 27 patients with one-stage tubeless percutaneous nephrolithotomy. Of the 3 patients with postoperative fever, 2 had the tubeless technique applied. One patient with 3 channels was given renal artery interventional embolization for control of postoperative bleeding. None of the 30 patients included in the study developed sepsis. The final stone-free rate was 93.3% (28/30) on repeat computed tomography at 1 month postoperatively. The final stone-free rate was 92.6% in the 27 patients undergoing one-stage tubeless percutaneous nephrolithotomy (25/27).

**Conclusions:**

One-stage tubeless PCNL is an available and safe option in carefully evaluated and selected calculous pyonephrosis patients.

## Background

Calculous pyonephrosis is a group of diseases in which urinary stones cause upper urinary tract obstruction, resulting in intrapelvic hypertension and subsequent purulent infection of the kidney. Upper urinary tract stone obstruction can lead to purulent urine infiltration into the renal parenchyma, causing impaired renal function, so relieving the obstruction and draining the pus is the key to treatment [1]. Staged treatment of calculous pyonephrosis can be chosen, i.e., adequate drainage and anti-infective treatment by placing a nephrostomy tube in the first stage. After the drained urine becomes clear and the bacterial culture is negative, second-stage lithotripsy via PCNL can be performed to prevent urogenic sepsis caused by bacterial and endotoxin reflux due to high perfusion pressure [2]. However, staged treatment of calculous pyonephrosis inevitably increases the length of the hospital stay and prolongs the duration of painful discomfort caused by indwelling nephrostomy tubes.

With the continuous development of percutaneous nephrolithotomy and further improvement of surgical instruments [3], the concept of one-stage percutaneous nephrolithotomy for calculous pyonephrosis has been accepted by urologists [1, 4–6]. A study by Huang et al. [7]. demonstrated that one-stage PCNL for calculous pyonephrosis reduced the length of hospital stay and treatment costs while ensuring patient safety. In addition, a meta-analysis by Xun et al. [8]. showed that tubeless PCNL reduced the patient’s hospital stay by 1.27 days, allowed for an earlier return to normal activity by 4.24 days, reduced the need for postoperative analgesia, and reduced the risk of urinary leakage compared to standard PCNL. Crook et al. [9] also showed that the tubeless PCNL technique significantly reduced postoperative pain and the length of hospital stay without increasing the incidence of postoperative complications. Therefore, it is reasonable to conclude that one-stage PCNL combined with the tubeless technique is feasible for the treatment of calculous pyonephrosis.

Since 2016, we have been practicing one-stage tubeless PCNL for carefully evaluated and selected patients with calculous pyonephrosis and have achieved good results so far, which are reported as follows.

## Methods

### Patients

We retrospectively analyzed the clinical data of 30 patients with a postoperative diagnosis of asymptomatic calculous pyonephrosis who were treated with PCNL in the Department of Urology, Affiliated Hospital of Jining Medical University, from January 2016 to January 2021. We defined asymptomatic calculous pyonephrosis as a patient diagnosed with a renal stone by computed tomography (CT), who did not require emergency surgery to remove the obstruction to protect their renal function, who had no preoperative diagnosis of a systemic inflammatory response syndrome, and who was observed intraoperatively to have turbid purulent fluid, pus accumulation in the kidney or pus moss attached to the stone. The patients’ general condition, laboratory parameters, imaging findings, and surgical data were obtained from our center’s electronic medical record database.

### Clinical data

Pre-, intra- and post-operative clinical data of the patients were collected. The preoperative data collected included imaging and laboratory findings in addition to baseline characteristics such as patient gender, comorbidities, and age. The results of the full ureteral CT were collected for ascertaining the location, type of stone (solitary stone, staghorn stone, multiple stones), size, CT value of the main part of the stone and the degree of hydronephrosis (mild, moderate, severe). Laboratory test data related to the infection were collected, including blood leukocytes, urine routine, urine bacterial culture and drug sensitivity tests. Patients with positive urine bacterial cultures were given antibiotics based on the drug sensitivity tests, and the rest were given empirical antibiotics. Surgical data were collected on the surgical procedure, operative time, and estimated intraoperative bleeding. Postoperative data were collected mainly on complications, immediate stone-free rate, and final stone-free rate.

The absence of residual stones or residual stones ≤ 4 mm was defined as stone-free [10]. CT was reviewed at 3–5 days and 1 month postoperatively, respectively, and was used to assess the patients’ immediate stone-free rate and final stone-free rate. The Clavien–Dindo grading system [11] was used to grade the patients for postoperative complications. A clinical diagnosis of sepsis was made for patients with a score of ≥ 2 on the sequential organ failure assessment (SOFA) scale [12].

### Surgical method

All 30 patients were given static inhalation compound anesthesia, and 20 mg of furosemide and 10 mg of dexamethasone sodium phosphate injection were given by intravenous push at the beginning of anesthesia. After the onset of anesthesia, 11 patients were placed in the truncated position, the F5 ureteral catheter was placed retrogradely, while the F16 catheter was left in the prone position; 19 patients were placed in the prone position after direct catheterization. Routine disinfection and towel laying were performed.

The best puncture site was selected between the mid-axillary line and the parascapular line on the affected side, and the target renal calyces were located under ultrasound (Mindray Portable M5, Shenzhen, China ) guidance. After the 18G puncture needle successfully entered the collecting system, the needle core was withdrawn, and white turbid purulent fluid was seen in some patients. For these patients, the pus was retained and sent for bacterial culture and drug sensitivity testing, and anti-infective treatment was performed with intravenous imipenem. A 0.038-inch zebra wire was introduced, the skin was incised at the puncture site for approximately 0.8 cm, and then the F20 fascial dilator was introduced using the one-shot dilatation method to establish percutaneous renal access. A F8.0/9.8 ureteroscope (Richard Wolf, Knittlingen, Germany) was inserted and connected to a suction device (a common negative pressure suction device used for blood suction during laparoscopic surgery) to aspirate the visible pus. After removing the purulent secretions, a holmium laser (Curestar HANS-H75, Hefei, China) and/or pneumatic ballistics (Funard, Beijing, China) were introduced for lithotripsy. Stone fragments were removed promptly during lithotripsy to avoid increased intrapelvic pressure due to stone obstruction between the sheaths.

For patients from which we could not remove the kidney stones with a single access, we performed multiple accesses stone removal. Patients who planned to undergo second-stage lithotripsy (1 case) or had 3 or more percutaneous renal accesses (2 cases) were treated with a postoperative indwelling nephrostomy tube to reduce the risk of infection. The remaining patients (27 cases) did not have indwelling nephrostomy tubes placed. All patients were operated on by the same surgeon.

### Statistical methods

We used Excel for data entry. SPSS 25.0 was used for data analysis. The measurement data are expressed as mean ± standard deviation. Count data are described using the number of cases.

## Results

In this study there were 8 men and 22 women. Seven patients had hypertensive disease and 4 had diabetes mellitus. The mean age of the patients was 53.8 (29–79) years with a mean body mass index 25.5 ± 5.2 kg/m^2^. CT findings throughout the ureter suggested that the stones were located in the right kidney in 12 cases and in the left kidney in 18 cases. There were single stones in 5 cases, staghorn stones in 9 cases and multiple stones in 16 cases; the CT value of the main part of the stones was 844.14 ± 340.51 HU; all were accompanied by different degrees of hydronephrosis. The mean preoperative blood leukocyte was 7.0 ± 2.5 (10^9^/L) in 30 patients.

The 11 patients with a positive urine culture in the middle segment were treated with antibiotics selected based on drug sensitivity tests. The other 19 patients with negative urine cultures were given empirical broad-spectrum antibiotics. The median duration of antibiotic use was 3 (1–10) days in all patients. The preoperative urine routine suggested 8 patients with positive nitrite, of which 7 had a positive urine culture (5 *Escherichia coli* and 2 *Klebsiella pneumoniae*). Patient-specific basic information and their stone characteristics are summarized in Table 1.

**Table 1:** Basic information and stone characteristics of the patients (n = 30)

Patient Information	Results
Age (years, mean ± SD)	53.8 ± 11.8
Gender [cases, (%)]	
Male	8/30 (26.7)
Female	22/30 (73.3)
BMI [kg/m^2^, cases, (%)]	
< 18.5	1/30 (3.3)
18.5–24.9	14/30 (46.7)
> 25.0	15/30 (50.0)
Underlying disease [cases, (%)]	
None	19/30 (63.3)
Hypertensive disease	7/30 (23.3)
Diabetes mellitus	4/30 (13.3)
Stone location [cases, (%)]	
Right	12/30 (40.0)
Left	18/30 (60.0)
Type of stone [cases, (%)]	
Solitary stone	5/30 (16.7)
Staghorn stone	9/30 (30.0)
Multiple stones	16/30 (53.3)
Length of single and staghorn stone (mm, mean ± SD)	29.6 ± 10.9
Multiple stones length diameter (mm, mean ± SD)	56.7 ± 19.1
CT values of stones [HU, cases, (%)]	
< 800	17/30 (56.7)
≥ 800	13/30 (43.3)
Hydronephrosis [cases, (%)]	
Mild	11/30 (36.7)
Moderate	11/30 (36.7)
Severe	8/30 (26.6)
Preoperative blood WBC (10^9^/L)	7.0 ± 2.5
Preoperative urine culture [cases, (%)]	
Negative	19/30 (63.3)
*Escherichia coli*	7/30 (23.3)
*Klebsiella pneumoniae*	2/30 (6.7)
*Pseudomonas aeruginosa*	1/30 (3.3)
*Aspergillus chimaera*	1/30 (3.3)
Preoperative urine nitrite [Example, (%)]	
Positive	8/30 (26.7)
Negative	22/30 (73.3)

*BMI* body mass index, *CT* computed tomography, *SD* standard deviation, *WBC* white blood cell

All 30 patients were successfully treated in one stage, with a mean operative time of 66.6 ± 34.7 min and a mean estimated intraoperative blood loss of 16.67 ± 14.34 mL. The mean postoperative hospital stay was 5.0 ± 3.1 days. The mean postoperative hospital stay was 4.6 ± 2.5 days for the 27 patients with one-stage tubeless percutaneous nephrolithotomy. Postoperative anti-infective treatment with imipenem was continued for 2–3 days for the 19 patients with severe pus accumulation in the kidney. The pus was cultured and 11 of them were positive, and antibacterial drugs were continued according to the results of the drug sensitivity test.

Postoperative infrared spectroscopy was used to determine the composition of the stones, and 14 cases were found to contain infected stones, while the remaining 15 cases had calcium oxalate as the main component and 1 case had anhydrous uric acid.

The mean postoperative blood leukocyte was 9.6 ± 4.3 (10^9^/L) in 30 patients. Three patients developed an elevated body temperature (> 38.5 °C) postoperatively, two of whom were patients who underwent one-stage tubeless percutaneous nephrolithotomy. As a Clavien–Dindo grade I complication, we continued to give antibacterial drugs and symptomatic treatment and all improved; one 3-channel patient developed postoperative hematuria and decreased hemoglobin to 70 g/L and was given renal artery interventional embolization (Clavien–Dindo grade IIIa), and the patient’s condition was controlled. None of the 30 patients included in this study developed sepsis.

The immediate stone-free rate and final stone-free rate were 83.3% (25/30) and 93.3% (28/30) in 30 patients when CT was repeated 3–5 days and 1 month after surgery, respectively. The immediate and final stone-free rates were 92.6% (25/27) for the 27 patients who did not have an indwelling nephrostomy tube. One of the patients with residual stones had an intraoperative nephrostomy tube placed because of the prolonged operation due to the stone filling in multiple calyces, and the stone was removed in a second stage operation 1 week later. Four patients had residual stones less than 10 mm in size, and 2 of them expelled them within 1 month after the operation. The other two patients without tubularization were treated with extracorporeal shock wave lithotripsy, and the stones were expelled successfully. The surgical results and postoperative complications of the patients are summarized in Table 2.

**Table 2:** Surgical outcomes and postoperative complications of the patients (n = 30)

Statistical variables	Results
Operative time (min, mean ± SD)	66.6 ± 34.7
Estimated intraoperative bleeding volume (mL, mean ± SD)	16.7 ± 14.3
Retained nephrostomy tube [cases, (%)]	
Yes	3/30 (10.0)
None	27/30 (90.0)
Postoperative hospital stay of all 30 patients (day, mean ± SD)	5.0 ± 3.1
Postoperative hospital stay of the 27 one-channel patients (day, mean ± SD)	4.6 ± 2.5
Pus culture [cases, (%)]	
Negative	8/19 (42.1)
*Aspergillus chimaera*	5/19 (26.3)
*Escherichia coli*	3/19 (15.8)
*Klebsiella pneumoniae*	2/19 (10.5)
*Pseudomonas aeruginosa*	1/19 (5.3)
Postoperative blood WBC (10^9^/L)	9.6 ± 4.3
Postoperative complications [cases, (%)]	
None	26/30 (86.7)
Body temperature > 38.5 °C	3/30 (10.0)
Hemorrhage	1/30 (3.3)
Sepsis, peripheral organ damage, death	0
Immediate stone-free rate in all 30 patients [cases, (%)]	25/30 (83.3)
Final stone-free rate in all 30 patients [cases, (%)]	28/30 (93.3)
Immediate stone-free rate in the 27 one-channel patients [cases, (%)]	25/27 (92.6)
Final stone-free rate in the 27 one-channel patients [cases, (%)]	25/27 (92.6)
Major stone composition [cases, (%)]	
Calcium oxalate stones	15/30 (50.0)
Infected stones	14/30 (46.7)
Anhydrous uric acid stones	1/30 (3.3)

## Discussion

Percutaneous nephrolithotomy has become the first choice for the treatment of complex stones [13], and postoperative drainage with a nephrostomy tube is routine. Bellman [14] first introduced tubeless PCNL, which means that no nephrostomy tube is left in place after surgery. The advantages of tubeless PCNL are mainly reflected in the obvious reduction of postoperative pain, the reduced need for analgesic drugs, and the shortening of the postoperative hospital stay and the time to return to normal activities. With the accumulation of surgical experience and the continuous development of surgical instruments, this procedure has been slowly accepted.

Previous treatment options for calculous pyonephrosis were based on staged surgery, and studies of one-stage PCNL for calculous pyonephrosis have also been reported, but there are no previous reports on the application of tubeless PCNL in the treatment of calculous pyonephrosis. We first proposed the concept of one-stage tubeless PCNL for the treatment of calculous pyonephrosis. However, this surgical option may be too aggressive for certain patients with obvious signs of infection. Therefore, in this study, we only share our experience of performing one-stage tubeless PCNL in patients with what we define as asymptomatic calculous pyonephrosis.

The establishment of percutaneous renal channels requires identification of the target calyces and precise puncture of the target based on preoperative CT and intraoperative ultrasound exploration. In patients with more than two percutaneous renal channels, there is a relative increase in damage to the renal parenchyma, which can disrupt the barrier function of the collecting system, introducing the possibility of bacteria and their toxins entering the bloodstream, increasing the risk of postoperative infection [15]. In this study, nephrostomy tubes were placed for adequate drainage in patients with three and four channels.

After establishing percutaneous renal access, the F8.0/9.8 ureteroscope was introduced and connected to a negative pressure suction device commonly used in the operating room to clean up the pus by negative pressure suction and fully expose the required field of view for lithotripsy, which is a simple, convenient, and economical method. Holmium laser and/or pneumatic ballast is applied for lithotripsy. During lithotripsy, a large number of bacteria are present within the stone and can enter the urinary collecting system. The operation time needs to be kept as short as possible, and for those found to have excessive stone load during lithotripsy, the operation needs to be conducted in two stages. After the first stage, we place a nephrostomy tube and then perform a second stage operation later.

Hoslt et al. [16] showed that the normal intrapelvic pressure is about 7.35 mmHg, and an intrapelvic pressure above 30 mmHg will lead to reflux of the renal pelvic veins and lymphatic vessels. Thus, attention needs to be paid to timely intraoperative cleaning of the debris accumulated in the peel-away sheath to avoid obstruction of the perfusate outflow. This can cause intrapelvic hypertension, which can cause reflux of bacteria and their toxins into the circulatory system, leading to the possibility of sepsis or even infectious shock [17].

An analysis of risk factors for the development of sepsis after calculous pyonephrosis surgery in one study found that intraoperative hypotension and emergency surgery were independent variables associated with intraoperative and postoperative urinary sepsis [1]. We believe that it is feasible to perform tubeless PCNL in patients with calculous pyonephrosis who have no obvious preoperative signs of infection, no massive intraoperative bleeding, no intraoperative kidney perforation or severe urinary extravasation, and no ureteral injury or obstruction. This is because it is more in line with the concept of rapid postoperative recovery while ensuring safety and effectiveness, and it also reduces the financial pressure on the patient. In this study, the mean postoperative length of stay was 4.6 ± 2.5 days in 27 patients with tubeless PCNL, which was increased to 5.0 ± 3.1 days by the addition of 3 additional patients with indwelling nephrostomy tubes. Although this study was not as rigorous as a randomized controlled trial, we believe it has some reference value. However, to ensure the successful implementation of this treatment option, the operator needs to have extensive surgical experience, to master the lithotripsy technique to shorten the operation time, and to use antimicrobial drugs appropriately.

In patients with renal pus due to upper urinary tract stone obstruction, studies have shown that the incidence of postoperative urogenital sepsis ranges from 0.3–4.7% [18]. To reduce the likelihood of serious infection, it is important to use antimicrobial drugs appropriately at all stages during the treatment process. In this study, 30 patients enrolled in the study had preoperative mid-stage urine cultures and only 11 were positive, which is consistent with Aron et al. [19], who reported that the preoperative rate of positive mid-stage urine cultures in patients with stony sepsis without signs of infection was < 50%. For patients with positive urine cultures, we administered sensitive antibiotics based on drug sensitivity tests, and all patients with negative urine cultures were selected for empirical anti-infective treatment with broad-spectrum antibacterial drugs. Preoperative urine routine suggested that 8 patients were positive for nitrite, and 5 of them had positive urine culture for *Escherichia coli*. Since *Enterobacteriaceae* can convert nitrate to nitrite [20], the possibility of *E. coli* infection is higher in patients with positive preoperative nitrite and requires vigilance.

In this study, 30 patients were given 20 mg of furosemide and 10 mg of dexamethasone sodium phosphate injection in conjunction with preoperative anesthesia. Furosemide can reduce the reflux and absorption of bacteria and their toxins due to intraoperative perfusion caused by intrapelvic hypertension by inhibiting renal tubular reabsorption and increasing the glomerular filtration rate. Dexamethasone has an anti-inflammatory and antipyretic effect, and the combination of the two may reduce the incidence of urogenic sepsis after PCNL [21]. We gave imipenem intraoperatively to patients with renal pus, and considering the higher risk of sepsis, we continued imipenem treatment for 2–3 days postoperatively and then chose antibiotics to continue treatment according to the patient’s drug sensitivity results. Although none of the patients in this study developed sepsis postoperatively, vigilance is required. Once a patient develops urogenital sepsis postoperatively, a combination of fluid resuscitation, anti-infection and removal of the source of infection is required[22].

One-stage tubeless PCNL was performed in 27 of the 30 patients included in our center, and two patients developed postoperative fever, which we considered to be related to surgical stress, and both improved after symptomatic treatment was given. The immediate stone-free rate was 92.6% on repeat CT from 3 to 5 days after surgery, which was similar to the rate previously reported in the literature [10], and the final stone-free rate remained at 92.6% after 1 month. Extracorporeal shock wave lithotripsy was given to the patients with residual stones and the stones were successfully expelled. This is an exploratory study and all procedures were performed by the same experienced operator in our center. Our findings have confirmed the effectiveness and safety of this surgical protocol.

To the best of our knowledge, this study is the first to propose the idea of one-stage tubeless percutaneous nephrolithotomy for the treatment of calculous pyonephrosis, and the safety and efficacy of tubeless PCNL were initially confirmed in this study. However, this study still has some limitations. First, this is a single-center retrospective study and the presence of selection bias is inevitable, making it difficult to interpret our results more internationally and avoid bias altogether. The small sample size is another disadvantage, which makes the results we care about (complications, stone-free rate, etc.) likely to be inaccurate. A multicenter, large randomized controlled trial of one-stage percutaneous nephrolithotomy versus one-stage tubeless percutaneous nephrolithotomy for the treatment of calculous pyonephrosis is necessary to further evaluate the advantages and disadvantages of tubeless PCNL in the context of the major drawbacks of this study.

## Conclusions

In conclusion, for patients with calculous pyonephropathy who had no obvious signs of infection before surgery and were accidentally discovered during surgery, one-stage tubeless percutaneous nephrolithotomy has good efficacy and safety.

## Data Availability

The raw datasets used and/or analyzed during the current study are available from the corresponding author on reasonable request.

## References

[1] Liang X, Huang J, Xing M (2019). Risk factors and outcomes of urosepsis in patients with calculous pyonephrosis receiving surgical intervention: a single-center retrospective study. BMC Anesthesiol.

[2] Türk C, Petřík A, Sarica K (2016). EAU guidelines on interventional treatment for urolithiasis. Eur Urol.

[3] Lai D, Xu W, Chen M (2020). Minimally invasive percutaneous nephrolithotomy with a novel vacuum-assisted access sheath for obstructive calculous pyonephrosis: a randomized study. Urol J.

[4] Wang J, Zhou DQ, He M (2013). One-phase treatment for calculous pyonephrosis by percutaneous nephrolithotomy assisted by EMS LithoClast master. Chin Med J (Engl).

[5] Zhou DQ, Wang J, Li WG (2009). Treatment of calculous pyonephrosis with percutaneous nephrolithotomy via the standard access. Nan Fang Yi Ke Da Xue Xue Bao.

[6] Chen L, Li JX, Huang XB, Wang XF (2014). Analysis for risk factors of systemic inflammatory response syndrome after one-phase treatment for apyrexic calculous pyonephrosis by percutaneous nephrolithotomy. Beijing Da Xue Xue Bao Yi Xue Ban.

[7] Huang J, Song L, Xie D (2016). A randomized study of minimally invasive percutaneous nephrolithotomy (MPCNL) with the aid of a patented suctioning sheath in the treatment of renal calculus complicated by pyonephrosis by one surgery. BMC Urol.

[8] Xun Y, Wang Q, Hu H (2017). Tubeless versus standard percutaneous nephrolithotomy: an update meta-analysis. BMC Urol.

[9] Crook TJ, Lockyer CR, Keoghane SR, Walmsley BH (2008). Totally tubeless percutaneous nephrolithotomy. J Endourol.

[10] Zhu H, Zhao Z, Cheng D (2021). Multiple-tract percutaneous nephrolithotomy as a day surgery for the treatment of complex renal stones: an initial experience. World J Urol.

[11] Mitropoulos D, Artibani W, Biyani CS, Bjerggaard Jensen J, Rouprêt M, Truss M (2018). Validation of the Clavien–Dindo grading system in urology by the European association of urology guidelines ad hoc panel. Eur Urol Focus.

[12] Singer M, Deutschman CS, Seymour CW (2016). The third international consensus definitions for sepsis and septic shock (Sepsis-3). JAMA.

[13] Girisha TD, Dev P, Vijaykumar R, Dharwadkar S, Madappa KM (2019). Single-step dilatation in percutaneous nephrolithotomy, its safety and efficacy: a prospective, single-center study. Urol Ann.

[14] Bellman GC, Davidoff R, Candela J, Gerspach J, Kurtz S, Stout L (1997). Tubeless percutaneous renal surgery. J Urol.

[15] Lai WS, Assimos D (2018). Factors associated with postoperative infection after percutaneous nephrolithotomy. Rev Urol.

[16] Holst U, Dissing T, Rawashdeh YF, Frokiaer J, Djurhuus JC, Mortensen J (2003). Norepinephrine inhibits the pelvic pressure increase in response to flow perfusion. J Urol.

[17] Kreydin EI, Eisner BH (2013). Risk factors for sepsis after percutaneous renal stone surgery. Nat Rev Urol.

[18] Seitz C, Desai M, Häcker A (2012). Incidence, prevention, and management of complications following percutaneous nephrolitholapaxy. Eur Urol.

[19] Aron M, Goel R, Gupta NP, Seth A (2005). Incidental detection of purulent fluid in kidney at percutaneous nephrolithotomy for branched renal calculi. J Endourol.

[20] Dadzie I, Quansah E, Puopelle Dakorah M, Abiade V, Takyi-Amuah E, Adusei R (2019). The effectiveness of dipstick for the detection of urinary tract infection. Can J Infect Dis Med Microbiol.

[21] Qi T, Qi X, Chen X, Jin X (2021). The retrospective study of perioperative application of dexamethasone and furosemide for postoperative anti-inflammation in patients undergoing percutaneous nephrolithotomy. Int Urol Nephrol.

[22] Naber KG, Bonkat G, Wagenlehner F (2020). The EAU and AUA/CUA/SUFU guidelines on recurrent urinary tract infections: what is the difference. Eur Urol.

